# Case Report: Uterine Anomalies in Girls With a Congenital Solitary Functioning Kidney

**DOI:** 10.3389/fped.2021.791499

**Published:** 2021-12-14

**Authors:** Mark J. C. M. van Dam, Bas S. H. J. Zegers, Michiel F. Schreuder

**Affiliations:** ^1^Department of Pediatrics, Máxima Medical Center, Veldhoven, Netherlands; ^2^Department of Pediatrics, Maastricht University Medical Center, Maastricht, Netherlands; ^3^Department of Pediatric Nephrology, Radboud University Medical Center, Radboud Institute for Molecular Life Sciences, Amalia Children's Hospital, Nijmegen, Netherlands

**Keywords:** congenital anomalies of the kidney and urinary tract, solitary functioning kidney, Müllerian anomalies, clinical management, case series

## Abstract

Unilateral renal agenesis and multicystic dysplastic kidney, resulting in a contralateral solitary functioning kidney (SFK), are part of the broad spectrum of congenital anomalies of the kidney and urinary tract (CAKUT). In girls with SFK, screening for asymptomatic Müllerian anomalies of uterus and vagina is not yet routinely performed, and therefore often overlooked until clinical complications in the menstrual cycle or fertility process occur. In this case series, we report on four teenagers with congenital SFK presenting with menstrual problems due to a Müllerian anomaly. Routine peri-menarchal screening for Müllerian anomalies in girls with SFK may provide timely counseling, surgical treatment and prevention of associated complications such as endometriosis, infertility and miscarriages.

## Introduction

Unilateral renal agenesis (URA) and multicystic dysplastic kidney (MCDK), resulting in a contralateral solitary functioning kidney (SFK), are part of the broad spectrum of congenital anomalies of the kidney and urinary tract (CAKUT). A SFK predisposes for the development of kidney injury, evidenced by albuminuria and/or hypertension, and chronic kidney disease (CKD) in later life, and patients with SFK should be followed throughout the entire lifespan (1). More than 50% of children with a SFK suffer from proteinuria or hypertension before they reach adulthood (2). Several factors have been suggested to select patients with a higher risk, such as additional CAKUT or lack of compensatory growth (3). The current guidelines advise regular follow-up with evaluation of growth, blood pressure, proteinuria, kidney function, and ultrasound of the kidney during childhood and maturity (4, 5).

URA is not only associated with other CAKUT, but also with extra-renal malformations, such as anomalies of the Müllerian system (6). Specifically the Herlyn-Werner-Wunderlich syndrome (HWWS), also known as OHVIRA (obstructed hemivagina with ipsilateral renal agenesis) syndrome, and Mayer-Rokitansky-Küster-Hauser syndrome are well-known entities (7, 8). This association can be explained by embryology: the vagina is formed by the interaction between the Müllerian tubercle and the mesonephric (or Wolffian) ducts. These mesonephric ducts induce the correct development and fusion of the Müllerian ducts to form the uterus. So, an anomaly of the Wolffian duct leads to renal agenesis or hypoplasia, uterine anomalies like uterus didelphys, and blind or atretic hemivagina, ipsilateral with renal agenesis (9). Other CAKUT, such as an ectopic kidney and horseshoe kidney can also be associated with Müllerian anomalies (10). In Table 1 the main Müllerian anomalies are described using the classification from the European Society of Human Reproduction and Embryology (ESHRE) and European Society of Gynecological Endoscopy (ESGE) (11). A classification from the American Society of Reproductive Medicine (ASRM) (formerly known as the American Fertility Society) does exist as well, in which segmental agenesis or hypoplasia, unicornuate uterus, uterus didelphys, bicornuate uterus, septate and arcuate uterus form the main classification (12). In addition to the malformations detailed in Table 1, one or both ovaries may be absent.

**Table 1:** Classification of female genital tract anomalies according to the European Society of Human Reproduction and Embryology (ESHRE) and European Society of Gynecological Endoscopy (ESGE).

**Uterine anomaly**		**Cervical anomaly**	**Vaginal anomaly**
**Main class**	**Sub-class**		
U0—normal uterus		C0—normal cervix	V0—normal vagina
U1—dysmorphic uterus	a. T-shaped b. Infantilis c. Others	C1—Septate cervix C2—Double ‘normal' cervix	V1—Longitudinal non-obstructing vaginal septum V2—Longitudinal obstructing vaginal septum
U2—Septate uterus	a. Partial b. Complete	C3—Unilateral cervical aplasia	V3—Transverse vaginal septum and/or imperforate hymen
U3—Bicorporeal uterus	a. Partial b. Complete c. Bicorporeal septate	C4—Cervical aplasia	V4—Vaginal aplasia
U4—Hemi-uterus	a. With rudimentary cavity (communicating or not horn) b. Without rudimentary cavity (horn without cavity/no horn)		
U5—Aplastic	a. With rudimentary cavity (bi- or unilateral horn) b. Without rudimentary cavity (bi- or unilateral uterine remnants/aplasia		
U6—Unclassified malformations			

Even though the coincidence of congenital SFK and Müllerian anomalies has been described in many cases and studies, screening girls with SFK for uterine and vaginal anomalies has only been described in a recent recommendation (5), but may not yet be implemented in routine clinical care. Diagnosis of Müllerian malformations is therefore often delayed until after menarche when affected girls present themselves with dysmenorrhea, intermenstrual bleeding, mucopurulent vaginal discharge, and/or abdominal pain as a result of obstructive hematocolpos (13). These, and other symptoms suspicious for Müllerian anomalies are presented in Table 2. Screening, early diagnosis, and timely treatment may prevent complications such as endometriosis (14), infertility, miscarriages and spontaneous abortion (15, 16).

**Table 2:** Overview of symptoms suspicious for a Müllerian anomaly in girls with a SFK.

• Primary amenorrhea with normal secondary sexual characteristics • Dysmenorrhea • Sparse menses, both regarding frequency or amount of blood loss • Cyclic/recurring/intermenstrual abdominal or pelvic pain • Mucopurulent vaginal discharge • In- or subfertility • Endometriosis • (Recurrent) miscarriages • Premature delivery • Dyspareunia

To emphasize the necessity to screen for Müllerian anomalies in girls with congenital SFK, we report on four girls who presented with uterine and vaginal complaints in their follow-up for SFK. In Table 3, data of these children are summarized.

**Table 3:** Summary of the four described cases.

	**Case 1**	**Case 2**	**Case 3**	**Case 4**
Cause of solitary functioning kidney	Agenesis of the left kidney associated with multiple congenital anomalies in the VACTERL spectrum	Agenesis of the left kidney	Multicystic dysplastic left kidney	Severe hydronephrotic or multicystic dysplastic left kidney
Symptoms suspicious for a Müllerian anomaly	Dysmenorrhea and recurring mild abdominal pain without intermenstrual bleeding	Progressive dysmenorrhea and intermenstrual abdominal pain	Irregular menses and inconstant vaginal blood loss	None. A routine abdominal ultrasound was suspicious for a Müllerian anomaly
Age of onset symptoms	13 years	11 years	11 years	Not applicable
Age of diagnosis	15 years	11 years	11 years	12 years
Type of Müllerian anomaly	Hemi-uterus with a non-communicating rudimentary, fluid-filled left uterine horn without anomalies of the cervix and vagina	Left-sided non-communicating unicornuate uterus and a ipsilateral hematosalpinx	Uterus didelphys without vaginal anomalies	Bicornuate uterus with two normally shaped cervices and a longitudinal obstructing vaginal septum

## Cases

### Case 1

First, we describe a girl, born after 34 weeks of gestation, with multiple congenital anomalies in the VACTERL spectrum: anal atresia, perimembranous ventricular septal defect with mild stenosis of the pulmonary arteries, butterfly and block vertebrae and agenesis of the left kidney. The neonatal period was complicated by a febrile E. coli urinary tract infection (UTI) in a dilated collecting system in the right-sided SFK, with transient acute kidney injury. Vesicoureteral reflux (VUR) was ruled out with a voiding cystourethrography (VCUG). After her menarche at the age of 13 years, she developed dysmenorrhea and recurring mild abdominal pain without intermenstrual bleeding. On routine follow-up of the SFK at the age of 15 years, her estimated glomerular filtration rate (eGFR, based on the bedside Schwartz equation) was 104 ml/min/1.73 m^2^ and there was no proteinuria or hypertension. Beside a normal looking solitary right kidney, measuring 11.3 cm in length, a bicornuate uterus was suspected on the abdominal ultrasound. The subsequent abdominal magnetic resonance imaging (MRI) revealed a hemi-uterus with a non-communicating rudimentary, fluid-filled left uterine horn without anomalies of the cervix and vagina. A partial hysterectomy was performed, and at the age of 17 years, she has a normal menstrual cycle without abdominal pain.

### Case 2

Second, we describe a full-term girl whose antenatal ultrasound was unable to visualize the left kidney and a SFK on the right side was confirmed after birth. Menarche occurred at the age of 11 years, and 6 months later she presented with progressive dysmenorrhea and intermenstrual abdominal pain. Abdominal ultrasound was suspect of a uterus didelphys with a single abdominal cyst, with a diameter of 10 cm. Subsequent MRI demonstrated a left-sided non-communicating unicornuate uterus and a ipsilateral hematosalpinx. She underwent a unilateral partial hysterectomy and salpingectomy, and oral contraceptives were started. At the age of 14 years, eGFR is 96 ml/min/1.73 m^2^, without proteinuria or hypertension. The ultrasound shows a solitary right kidney of 13.0 cm.

### Case 3

The third case concerns a girl whose antenatal ultrasound at 20 weeks pregnancy suggested 2 kidneys. However, at the subsequent ultrasound at 32 weeks pregnancy the left kidney was no longer visible. Post-natal ultrasound demonstrated a solitary right-sided kidney with hydronephrosis and dilatation of the calices. A VCUG and mercaptoacetyltriglycine (MAG3) renography ruled out VUR and ureteropelvic-junction (UPJ) stenosis, respectively. At the age of 11 years, menarche occurred after which menses were sparse, both in frequency and in the amount of blood loss. Because of irregular menses and inconstant vaginal blood loss the gynecologist was consulted. The abdominal ultrasound was suspect of an uterine anomaly and the subsequent MRI revealed a uterus didelphys without vaginal anomalies. Currently she is 12 years old, has started oral contraceptives and is awaiting surgical correction at the age of 16 years. eGFR is 101 ml/min/1.73 m^2^, proteinuria and hypertension are absent, and ultrasound shows a solitary right kidney of 11.0 cm.

### Case 4

Finally, we describe a full term-born girl whose antenatal ultrasound suggested a cyst in the left kidney. On post-natal ultrasound, the left kidney was enlarged, with severe dilatation of both renal pelvis and calices, while the anatomically normal right kidney was mildly enlarged. The suspected compensatory hypertrophy of the right kidney was confirmed by the MAG3 renography, which revealed an afunctional left kidney. The VCUG was unremarkable. At the age of 12 years old, an abdominal ultrasound was suspect of a bicornuate uterus with significant vaginal fluid stasis. Menarche had not yet occurred, nor vaginal blood loss or (periodic) abdominal pain. Abdominal MRI confirmed the presence of a bicornuate uterus with two normally shaped cervices and a longitudinal obstructing vaginal septum. She underwent an uncomplicated resection of the vaginal septum. Currently, her eGFR is 109 ml/min/1.73 m^2^, she has no proteinuria or hypertension, and ultrasound shows a solitary right kidney of 14.5 cm.

## Discussion

Regardless of etiology, a SFK predisposes for the development of glomerular hyperfiltration injury (hypertension and/or proteinuria), with long-term chronic kidney disease (1). Therefore, clinical practice recommendations state that children with a SFK should be regularly evaluated for blood pressure, albuminuria/proteinuria, kidney function and size. Even though the coincidence of congenital SFK and Müllerian anomalies has been described in many cases and studies, screening girls with SFK for uterine and vaginal anomalies has only been described in a recent recommendation (5).

The estimated incidence of URA is 1 in 2,000 children, and it is estimated that 11–30% of the girls with URA has a female tract anomaly (6, 16). So far, there is insufficient data concerning the underlying genetics of SFK and Müllerian anomalies, despite the ever increasing knowledge in this area (17). Although the gold standard for detecting Müllerian anomalies is MRI (18), transabdominal ultrasound is commonly employed as the first modality for the initial workup of Müllerian anomalies, as it is simple, low-invasive and low-cost. Moreover, performing abdominal ultrasound is already part of the recommended follow-up in children with SFK, and thus easy to implement in clinical practice. However, the accuracy of transabdominal ultrasound in screening for Müllerian anomalies is highly dependent on the experience of the examiner: in a single-center study among 22 girls with a mean age of 12.2 ± 4.1 years the type of anomaly was characterized in only 59% of the children (19). The optimal timing to perform an abdominal ultrasound for detection of Müllerian anomalies is either in the first few weeks of life, when maternal hormonal stimulation is present, or in the pubertal period after the onset of thelarche (15, 19). Alternative diagnostics to detect Müllerian anomalies, such as hysterosalpingography and transvaginal pelvic sonography are not preferred over abdominal ultrasound or MRI in the pediatric population. Three-dimensional ultrasound can be considered as an alternative, though this requires a more specialized setting (20).

When a congenital SFK is detected in a girl, parents should be informed about the coincidence of congenital SFK and Müllerian anomalies. Though physicians must consider the emotional stress patients and their parents may experience after this announcement, the potential benefits of early diagnosis justify this counseling in clinical practice. Moreover, if a Müllerian anomaly is detected, physicians should focus on the psychological consequences of having abnormal internal genitalia. Abdominal ultrasound with a focus on Müllerian anomalies should be implemented in the routine clinical practice recommendations in the first weeks of life and/or in the pubertal period after the onset of thelarche. Besides this, physicians should specifically evaluate teenage girls with congenital SFK at the post-thelarche period for dysmenorrhea, intermenstrual bleeding, mucopurulent vaginal discharge, or cyclic abdominal pain among others. We suggest to perform a pelvic MRI in those girls with abnormal transabdominal ultrasound in the post-thelarche period, and/or in girls with specific complaints fitting within the spectrum of Müllerian anomalies. Implementing this screening with early diagnosis and subsequent timely treatment of Müllerian anomalies might decrease or even prevent complications such as endometriosis (14), infertility, miscarriages and spontaneous abortion (15, 16).

In conclusion, we advise peri-menarchal screening for potential Müllerian anomalies in girls with congenital SFK with attention for specific complaints and routine abdominal ultrasound focused on internal genitalia. Early detection of uterine and vaginal anomalies provides the possibility to timely counsel the patient and her family about the anomaly and treatment options to prevent menstrual complaints and fertility complications.

## Data Availability Statement

The original contributions presented in the study are included in the article/supplementary material, further inquiries can be directed to the corresponding authors.

## Author Contributions

Material preparation, data collection, and analysis were performed by MD and SZ. The first draft of the manuscript was written by MD. MD, SZ, and MS commented on previous versions of the manuscript, contributed to the study conception, and design. All authors read and approved the final manuscript.

## Conflict of Interest

The authors declare that the research was conducted in the absence of any commercial or financial relationships that could be construed as a potential conflict of interest.

## Publisher's Note

All claims expressed in this article are solely those of the authors and do not necessarily represent those of their affiliated organizations, or those of the publisher, the editors and the reviewers. Any product that may be evaluated in this article, or claim that may be made by its manufacturer, is not guaranteed or endorsed by the publisher.
